# Engineering Flocculation for Improved Tolerance and Production of d-Lactic Acid in *Pichia pastoris*

**DOI:** 10.3390/jof9040409

**Published:** 2023-03-27

**Authors:** Kittapong Sae-Tang, Pornsiri Bumrungtham, Wuttichai Mhuantong, Verawat Champreda, Sutipa Tanapongpipat, Xin-Qing Zhao, Chen-Guang Liu, Weerawat Runguphan

**Affiliations:** 1 National Center for Genetic Engineering and Biotechnology, 113 Thailand Science Park, Paholyothin Road, Klong 1, Klong Luang, Pathum Thani 12120, Thailand; 2 State Key Laboratory of Microbial Metabolism, Joint International Research Laboratory of Metabolic & Developmental Sciences, School of Life Sciences and Biotechnology, Shanghai Jiao Tong University, Shanghai 200240, China

**Keywords:** cell flocculation, d-lactic acid production, stress tolerance, yeast, *Pichia pastoris*

## Abstract

d-lactic acid, a chiral organic acid, can enhance the thermal stability of polylactic acid plastics. Microorganisms such as the yeast *Pichia pastoris*, which lack the natural ability to produce or accumulate high amounts of d-lactic acid, have been metabolically engineered to produce it in high titers. However, tolerance to d-lactic acid remains a challenge. In this study, we demonstrate that cell flocculation improves tolerance to d-lactic acid and increases d-lactic acid production in *Pichia pastoris*. By incorporating a flocculation gene from *Saccharomyces cerevisiae* (*ScFLO1*) into *P. pastoris* KM71, we created a strain (KM71-ScFlo1) that demonstrated up to a 1.6-fold improvement in specific growth rate at high d-lactic acid concentrations. Furthermore, integrating a d-lactate dehydrogenase gene from *Leuconostoc pseudomesenteroides* (*Lp*DLDH) into KM71-ScFlo1 resulted in an engineered strain (KM71-ScFlo1-LpDLDH) that could produce d-lactic acid at a titer of 5.12 ± 0.35 g/L in 48 h, a 2.6-fold improvement over the control strain lacking *ScFLO1* expression. Transcriptomics analysis of this strain provided insights into the mechanism of increased tolerance to d-lactic acid, including the upregulations of genes involved in lactate transport and iron metabolism. Overall, our work represents an advancement in the efficient microbial production of d-lactic acid by manipulating yeast flocculation.

## 1. Introduction

Petroleum-based routes for the production of platform chemicals and fuels are a large contributor to global greenhouse gas emissions [1,2]. An alternative approach is to produce these chemicals using renewable sources. Researchers have made advances in this field, including in the production of organic acids, bioplastic monomers, higher alcohols, and fatty alkanes [3,4,5,6,7,8]. Lactic acid has various applications in industries, such as in food production and plastic manufacturing [9,10]. In plastic production, lactic acid is used to make biocompatible and biodegradable poly(lactic acid) (PLA) plastics as a substitute for non-biodegradable petroleum-based plastics. To create PLA, the lactic acid monomer is polymerized. The mechanical and thermal properties of the resulting PLA are influenced by the optical purity of the lactic acid, which can be either the d-form, the l-form, or a mixture of both [11]. Blending poly-d-lactic acid with poly-l-lactic acid through a process known as stereocomplexation results in plastics with improved thermal stability [12,13]. The large-scale production of l-lactic acid by major chemical companies using microbes is well-established [14]. However, limited research exists on the production of d-lactic acid using microbes, creating a need for a more efficient method of producing this acid.

In nature, bacteria such as those found in the genera *Lactobacillus, Sporolactobacillus*, and *Leuconostoc* are efficient producers of d-lactic acid [10]. For instance, *Sporolactobacillus inulinus* YBS1-5 produced 107.2 g/L of d-lactic acid through fed-batch fermentation using corncob residue hydrolysates, with a yield of 0.85 g/g glucose [15]. Another example is *Leuconostoc mesenteroides* B512, which produced 60.2 g/L of d-lactic acid through shake-flask fermentation using sugarcane juice, with a yield of 0.51 g/g glucose [16]. 

In addition to these natural d-lactic acid producers, some industrial hosts without the natural ability to produce d-lactic acid have been genetically engineered to do so with high efficiency. One such example is *Pichia pastoris* (recently reclassified as *Komagataella phaffii*), a methylotrophic yeast that has been genetically modified for many years to produce various industrial enzymes, including phytase, hepatitis B surface antigen, and human serum albumin [17]. More recently, *P. pastoris* has been proven to be a highly productive producer of chemicals such as higher alcohols isobutanol and isopentanol, fragrant terpenoid (+)-nootkatone, and antioxidant lycopene [18,19,20,21,22]. Furthermore, *P. pastoris* is an appealing host for consolidated the bioprocessing of chemicals from biomass because of its efficiency in expressing biomass-degrading enzymes [23].

In an early study, de Lima and colleagues introduced the gene encoding l-lactate dehydrogenase from *Bos taurus* into *P. pastoris*, resulting in a strain that can produce l-lactic acid from glycerol at a yield of 0.236 g/g glycerol [24]. To improve the yield, the authors overexpressed the endogenous lactate transporter (PAS) and the final engineered strain produced l-lactic acid at a yield of 0.673 g/g glycerol and a titer of almost 30 g/L. More recently, Yamada and colleagues used *P. pastoris*’ ability to use methanol as the sole carbon source to produce d-lactic acid from methanol [25]. Specifically, the authors integrated multiple copies of the *Leuconostoc mesenteroides* d-lactate dehydrogenase gene into the non-transcribed spacer of the ribosomal DNA (rDNA) locus of *P. pastoris*. This resulted in a strain that produced d-lactic acid at a titer of 3.48 g/L directly from methanol, which corresponds to a yield of 0.22 g/g of methanol consumed.

While the mechanisms behind lactic acid tolerance and its impact on lactic acid production have been extensively studied in other microorganisms, particularly in the model yeast *S. cerevisiae* [26], so far, no study has been reported on organic acid tolerance in *P. pastoris*. It would be desirable to develop robust *P*. *pastoris* strains for efficient lactic acid production.

In the previous study, yeast cell flocculation was revealed to be associated with improved stress tolerance. For example, elevated acetic acid tolerance was reported in an industrial yeast, *S. cerevisiae* SPSC01, when compared to its non-flocculating mutant strain, where the flocculin gene *FLO1* was deleted [27]. In another report, flocculation was found to be related to furfural tolerance [28]. It is of interest whether flocculation also exerts any effect on the acid tolerance of *P. pastoris.*

In this study, we seek to investigate the inhibitory effect of high concentrations of d-lactic acid on the growth of *P. pastoris* and to enhance the yeast’s tolerance to d-lactic acid through engineered flocculation. Our ultimate goal was to advance the efficient microbial production of d-lactic acid.

## 2. Materials and Methods

### 2.1. Media, Strains, and Transformation

The yeast strains were constructed from *P. pastoris* KM71 (Invitrogen), as shown in Table 1. The plasmids were derived from the pGAPHisKlARS and pGAPHyg vectors [18,29], as well as the commercially available pGAPZ vector (Invitrogen). The primers used are listed in Table S1. Transformation of yeast was carried out using an electroporator, as previously described [19]. The electroporation parameters used were 1.5 kV, 25 μF, and 200 Ω. The plasmids were digested with the restriction enzyme *Avr*II prior to electroporation. Transformants were confirmed through colony PCR and DNA sequencing. *E. coli* DH5-alpha, which was used during plasmid construction and propagation, was grown in Luria–Bertani (LB) medium supplemented with the antibiotics hygromycin (100 μg/L), ampicillin (100 μg/L) or zeocin (100 μg/L) as necessary. *P. pastoris* was grown in YPD medium (10 g/L yeast extract, 20 g/L Bacto Peptone, and 20 g/L glucose) supplemented with the antibiotics zeocin (100 μg/L) or hygromycin (200 μg/L) as required. Yeast transformants harboring the HIS4 selectable marker were selected on a yeast minimal medium (MGY; 0.1 M phosphate buffer pH 6.0, yeast nitrogen base without amino acids (13.6 g/L), glycerol (20 g/L), d-biotin (0.4 mg/L), and thiamine-hydrochloride (133.3 mg/L)).

### 2.2. Plasmid Construction

The plasmids used in this study were generated from the pGAPZ_A (Thermo Fisher Scientific, Waltham, MA, USA), pGAPHis4 and pGAPHyg vectors [18]. These plasmids utilized the constitutive promoter from the glyceraldehyde-3-phosphate dehydrogenase gene (P_GAP_). The cloning required to construct the plasmids used in this study was performed using *E. coli* DH5α. Polymerase chain reaction (PCR) amplification was carried out using Phusion High-Fidelity polymerase (Thermo Fisher Scientific) following the manufacturer’s protocol. Briefly, the PCR was set under conditions of 98 °C for 30 s, a denaturation at 98 °C for 10 s, an annealing at the primers’ melting temperature (T_m_) + 3 °C for 30 s, and an extension at 72 °C for 15 s/kb of the expected PCR product. PCR was performed for 30 cycles, followed by a final extension at 72 °C for 5 min. The PCR fragments were then gel-purified using the QIAquick Gel Extraction Kit (Qiagen, Düsseldorf, Germany). Plasmids were constructed using enzyme ligation or homologous recombination in yeast, as described below. Enzyme ligation was carried out using T4 DNA Ligase (Thermo Fisher Scientific) at 22 °C for 1 h followed by heat inactivation of the enzyme at 65 °C for 10 min.

*Plasmid pGAPHis4-ScFlo1:* The *FLO1* gene was amplified from the genomic DNA of *S. cerevisiae* S288C using primers Flo1-hom-F and Flo1-hom-R. The PCR band was purified and then added to the purified *Not*I/*Apa*I-digested pGAPHis*Kl*ARS fragment to form pGAPHis4-ScFlo1 via homologous recombination in *S. cerevisiae*.

*Plasmid pGAPHyg-LpDldh:* The codon-optimized d-lactate dehydrogenase gene from *Leuconostoc pseudomesenteroides* BCC38023 (*LpDLDH*, GenBank accession number: MW574953) was obtained from the pUC57-LpDLDH plasmid [2]. The *LpDLDH* gene was amplified using primers LpDLDH-F and LpDLDH-R. The purified PCR band was ligated to the KpnI/NotI site of pGAP-hyg to yield pGAPHyg-LpDldh.

*Plasmid pGAPZ-PpJEN1:* The putative lactate transporter *PpJEN1* (homolog of *S. cerevisiae JEN1*; accession number XM_002492622.1) was amplified from the genomic DNA of *P. pastoris* KM71 using primers PpJEN1-F and PpJEN1-R. The purified PCR band was ligated to the EcoRI/NotI site of the pGAPZ plasmid to yield pGAPZ-PpJen1.

*Plasmid pGAPZ-PpADY2-1:* The putative acetate transporter *PpADY2-1* (homolog of *S. cerevisiae ADY2*; accession number XM_002491529.1) was amplified from the genomic DNA of *P. pastoris* KM71 using primers PpADY2-1-F and PpADY2-1-R. The purified PCR band was ligated to the EcoRI/NotI site of the pGAPZ plasmid to yield pGAPZ-PpADY2-1.

*Plasmid pGAPZ-PpADY2-2:* The putative acetate transporter *PpADY2-2* (homolog of *S. cerevisiae ADY2*; accession number XM_002489992.1) was amplified from the genomic DNA of *P. pastoris* KM71 using primers PpADY2-2-F and PpADY2-2-R. The purified PCR band was ligated to the EcoRI/NotI site of the pGAPZ plasmid to yield pGAPZ-PpADY2-2.

*Plasmid pGAPZ-PpFPS1:* The putative channel-like protein *PpFPS1* (homolog of *S. cerevisiae FPS1*; accession number XM_002494184.1) was amplified from the genomic DNA of *P. pastoris* KM71 using primers PpFPS1-F and PpFPS1-R. The purified PCR band was ligated to the EcoRI/NotI site of the pGAPZ plasmid to yield pGAPZ-PpFPS1.

*Plasmid pGAPZ-PpAcXp:* The putative transporter required for normal sporulation (accession number XM_002491528.1) was amplified from the genomic DNA of *P. pastoris* KM71 using primers PpAcXp-F and PpAcXp-R. The purified PCR band was ligated to the EcoRI/NotI site of the pGAPZ plasmid to yield pGAPZ-PpAcXp.

*Plasmid pGAPZ-PpFeRed:* The putative ferric reductase (accession number XM_002493603.1) was amplified from the genomic DNA of *P. pastoris* KM71 using primers PpFeRed-F and PpFeRed-R. The purified PCR band was ligated to the EcoRI/NotI site of the pGAPZ plasmid to yield pGAPZ-PpFeRed.

*Plasmid pGAPZ-PpFerXp:* The putative Ferrioxamine B transporter (accession number XM_002492843.1) was amplified from the genomic DNA of *P. pastoris* KM71 using primers PpFerXP-F and PpFerXP-R. The purified PCR band was ligated to the EcoRI/NotI site of the pGAPZ plasmid to yield pGAPZ-PpFerXP.

*Plasmid pGAPZ-PpLAFeXp:* The putative low-affinity Fe(II) transporter of the plasma membrane (accession number XM_002492163.1) was amplified from the genomic DNA of *P. pastoris* KM71 using primers PpLAFeXp-F and PpLAFeXp-R. The purified PCR band was ligated to the EcoRI/NotI site of the pGAPZ plasmid to yield pGAPZ-PpLAFeXp.

*Plasmid pGAPZ-PpFeO2OR:* The putative Ferro-O2-oxidoreductase (accession number XM_002491680.1) was amplified from the genomic DNA of *P. pastoris* KM71 using primers PpFeO2OR-F and PpFeO2OR-R. The purified PCR band was ligated to the EcoRI/NotI site of the pGAPZ plasmid to yield pGAPZ-PpFeO2OR.

*Plasmid pGAPZ-PpLAZnXp:* The putative low-affinity zinc transporter of the plasma membrane (accession number XM_002493905.1) was amplified from the genomic DNA of *P. pastoris* KM71 using primers PpLAZnXp-F and PpLAZnXp-R. The purified PCR band was ligated to the EcoRI/NotI site of the pGAPZ plasmid to yield pGAPZ-PpLAZnXp.

*Plasmid pGAPZ-PpH^+^-ATPase:* The putative plasma membrane H^+^-ATPase (accession number XM_002489588.1) was amplified from the genomic DNA of *P. pastoris* KM71 using primers PpHATPase-F and PpHATPase-R. The purified PCR band was ligated to the XhoI/NotI site of the pGAPZ plasmid to yield pGAPZ-PpH^+^-ATPase.

*Plasmid pGAPZ-PpSUR1:* The putative catalytic subunit of a mannosylinositol phosphorylceramide (MIPC) synthase (homolog of *S. cerevisiae SUR1*; accession number XM_002489517.1) was amplified from the genomic DNA of *P. pastoris* KM71 using primers PpSUR1-F and PpSUR1-R. The purified PCR band was ligated to the EcoRI/NotI site of the pGAPZ plasmid to yield pGAPZ-PpSUR1.

*Plasmid pGAPZ-PpHAZnXp:* The putative high-affinity zinc transporter of the plasma membrane (accession number XM_002492699.1) was amplified from the genomic DNA of *P. pastoris* KM71 using two sets of primers: PpHAZnXp-up-F and PpHAZnXp-up-R, and PpHAZnXp-dw-F and PpHAZnXp-dw-R. The two DNA fragments were assembled by overlap extension PCR (OE-PCR) and ligated to the EcoRI/NotI site of the pGAPZ plasmid to yield pGAPZ-PpHAZnXp.

### 2.3. Quantifying Production of d-Lactic Acid and Other Metabolites

The quantification of d-lactic acid and other metabolites was performed as previously described, with some modifications [2,30]. The engineered strains were prepared by pre-culturing 5 mL aliquots in a yeast minimal medium (MGYH; 0.1 M phosphate buffer pH 6.0, yeast nitrogen base without amino acids (13.6 g/L), glycerol (20 g/L), d-biotin (0.4 mg/L), thiamine-hydrochloride (133.3 mg/L), and l-histidine (20 mg/L)). The overnight cultures were used to inoculate 10 mL of the production medium (MGYH-glu; 0.1 M phosphate buffer pH 6.0, yeast nitrogen base without amino acids (13.6 g/L), glucose (100 g/L), d-biotin (0.4 mg/L), thiamine-hydrochloride (133.3 mg/L), and l-histidine (20 mg/L)) in 50 mL Corning tubes to reach an OD_600_ of 0.05. The cultures were incubated at 30 °C and 250 rpm, and samples were taken at 24, 48 and 72 h to measure OD_600_, d-lactic acid and extracellular metabolites.

High-performance liquid chromatography (HPLC) was used to measure the amount of d-lactic acid and other fermentation metabolites, including ethanol, glycerol, pyruvate, acetate and glucose, as previously described [2,30]. One mL of yeast culture was passed through a 0.2-micron nylon syringe filter (Filtrex, Bangkok, Thailand). The filtered sample was analyzed on an Agilent 1100 series HPLC with an Aminex HPX-87H ion-exchange column (Bio-Rad, Hercules, CA, USA). The parameters for the column were: 7.8 mm internal diameter, 300 mm length, and 9 μm particle size. The analysis was conducted using a 5 mM solution of H_2_SO_4_ as solvent with a flow rate of 0.68 mL/min for 30 min, with the column temperature kept at 60 °C. Metabolites were detected using Agilent 1200 series DAD and RID detectors. Two sets of HPLC standards were prepared. The first set contained the organic acids (pyruvate, prepared by using sodium pyruvate (Fluka, Morris Plains, NJ, USA) and acetate, prepared by using sodium acetate (Merck, Rahway, NJ, USA)), glycerol (Carlo Erba, Emmendingen, Germany), and ethanol (Supelco, St. Louis, MO, USA), while the second set contained d-glucose (Milipore, Burlington, MA, USA) and d-lactic acid (Sigma-Aldrich, St. Louis, MO, USA). All statistical analysis was performed using a two-tail, unpaired, heteroscedastic Student’s *t*-test.

### 2.4. RNA Isolation and Transcriptomics Analysis

The strains were grown overnight in 5 mL aliquots of MGYH medium, then used to inoculate 10 mL of MGYH medium in 50 mL Corning tubes to reach an initial OD_600_ of 0.05. The cultures were incubated at 30 °C and 250 rpm. After approximately 21 h, when the strains reached the mid-exponential phase, a 2 mL sample of each culture was collected, centrifuged for 5 min at 3000× *g*, and washed with 5 mL distilled water. Total RNA was extracted using the RNeasy Kit (Qiagen), and contaminating genomic DNA was removed by DNaseI digestion (Thermo Fisher Scientific) following the manufacturer’s protocol. RNA Integrity Numbers (RIN) were obtained using an Agilent 2100 Bioanalyzer (Agilent Technologies, Santa Clara, CA, USA), and all purified RNA samples had an RIN number higher than 8.0. RNA samples were kept at −80 °C until transcriptomics analysis. The RNA was sequenced by NovogeneAIT (Tianjin, China) using the Illumina NovaSeq PE150 platform, generating expression libraries of 150 nt read length. To avoid random errors, triplicate cultures were sampled. Raw PE reads underwent quality control via FASTP by taking out ambiguous bases (N base) and low-quality sequences (Q score < 20) [31]. The clean reads were mapped against the *P. pastoris* GS115 genome (GenBank assembly accession number: GCA_000027005.1) using HISAT2 [32], and read counts for each gene were calculated by HTSeq-count using SAM files and genome annotation files as inputs [33]. The number of uniquely mapped reads was then used to calculate each gene’s normalized expression values. Differentially expressed genes were identified using the DESeq2 package, based on raw counts of reads mapping to unique genes [34]. Genes with a fold change >2 and DESeq P_-adj_ value < 0.05 were considered to be expressed with significant differences between the control strain (KM71-LpDLDH) and the flocculation strain (KM71-ScFlo1-LpDLDH). All raw reads were deposited in NCBI with the accession number GSE224837.

### 2.5. Real-Time PCR

Total RNA was extracted and purified from the samples as described in the above section. cDNA was generated using RevertAid Reverse Transcriptase (Thermo Fisher Scientific) and following the manufacturer’s protocol. The relative expression levels of *PpJEN1, PpADY2-1, PpADY2-2, PpFeRed, PpAcXP, PpLAZnXp, PpHAZnXP, PpFPS1, PpFerXP, PpSUR1, PpH^+^-ATPase, PpFeO2OR, PpLAFeXp* and *LpDLDH* were quantified on a CFX96 Touch Real-time PCR Detection System (Biorad) using iQ SYBR Green Kit (Biorad). The real-time PCR parameters used were as follows: a polymerase activation at 95 °C for 3 min, a denaturation at 95 °C for 15 s, and an annealing/extension at 60 °C for 30 s, followed by a plate read. The PCR was performed for 40 cycles, which was followed by a melt curve analysis by ramping up the temperature from 60 °C to 95 °C at a 0.5 °C increment with 5 s/step. The RT-PCR was performed in triplicate, and the amount of total mRNA in all samples was normalized using *PpACT1*, a gene that encodes actin.

## 3. Results and Discussion

### 3.1. Expression of the Flocculation Protein Flo1 in P. pastoris Improves d-Lactic Acid Tolerance

The mechanisms behind lactic acid tolerance and how they impact lactic acid production have been extensively studied in other microorganisms, particularly in the model yeast *S. cerevisiae* [26]. However, little has been reported on the mechanism of organic acid tolerance in *P. pastoris*. In this work, we sought to determine whether flocculation can improve the yeast’s tolerance to lactic acid. Yeast flocculation is a reversible and nonsexual process by which yeast cells aggregate and form clumps known as flocs [35]. The result of flocculation is the rapid sedimentation of the flocs from the medium. This property is desirable in industrial yeasts as it enables easy separation from fermentation mashes, without the need for expensive equipment [36,37,38,39].

Flocculation mechanisms have been extensively studied in *S. cerevisiae*, but research in other yeast species is limited [35,40,41]. The process of flocculation involves the interaction of flocculins, a type of lectin-like protein, with receptors on neighboring cell walls, forming aggregates [42,43]. Factors such as pH, calcium ions, and organic stress also influence flocculation. The *FLO1*, *FLO5*, *FLO9*, *FLO10*, *FLO11*, *FLONL*, *FLONS*, and *Lg-FLO* genes have been identified as contributing to flocculation in *S. cerevisiae* [44,45,46]. These genes confer cell–cell adhesion, with the exception of *ScFLO11*, which is responsible for substrate adhesion. Flocculation mechanisms in other yeast species are less well understood, but also thought to be lectin-based [47,48,49].

**Figure 1:**
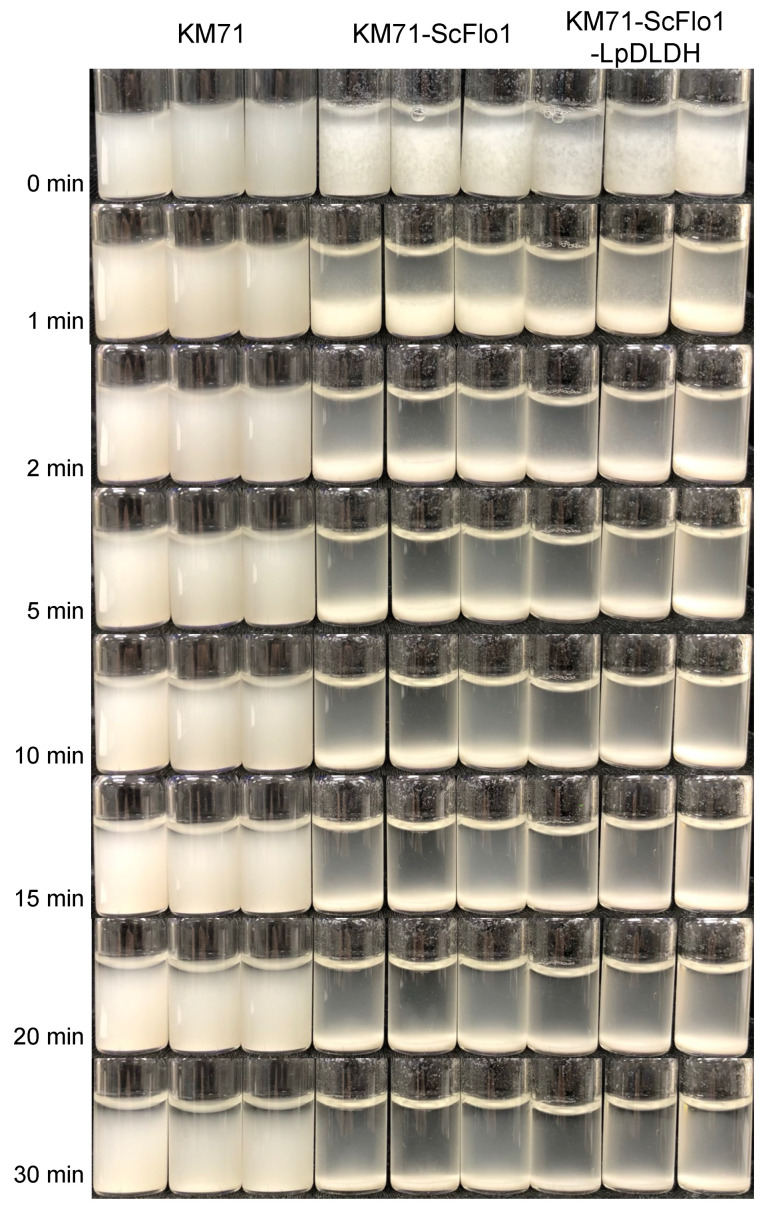
The flocculation phenotypes of engineered strains generated in this study. *P. pastoris* KM71, KM71-ScFlo1, and KM71-ScFlo1-LpDLDH were cultivated in MGYH-glu medium overnight at 30 °C at 250 rpm. One milliliter aliquots of the overnight cultures were transferred to 1.5 mL glass vials. The experiments were performed in biological triplicate.

**Figure 2:**
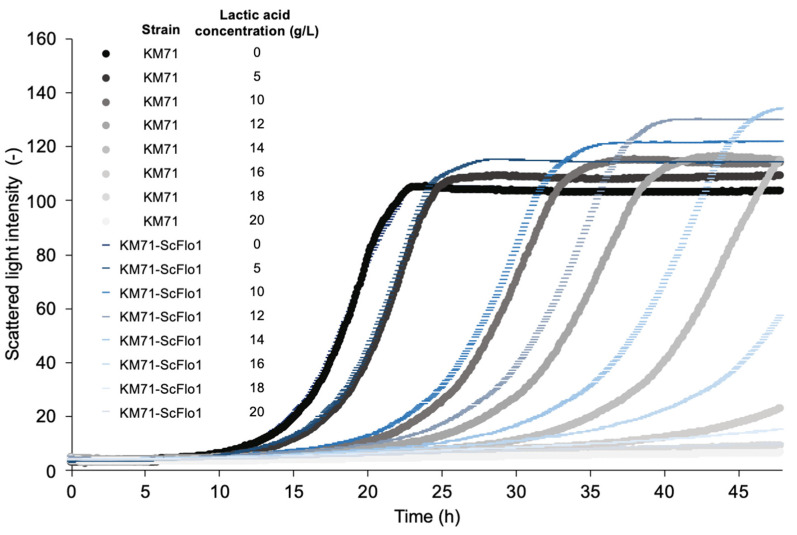
Effects of various lactic acid concentrations in YPD medium (liquid culture) on the growth of *P. pastoris* with or without *ScFlo1* overexpression. *P. pastoris* KM71 and KM71-ScFlo1 were cultivated in YPD medium with different concentrations (0, 5, 10, 12, 14, 16, 18, and 20 g/L) of lactic acid in a microflower plate. The cells were grown in the BioLector microfermenter (m2p labs) at 30 °C at 1100 rpm with 70% humidity for 48 h. Plates were sealed with porous film to minimize evaporation. The experiments were performed in biological triplicate.

**Figure 3:**
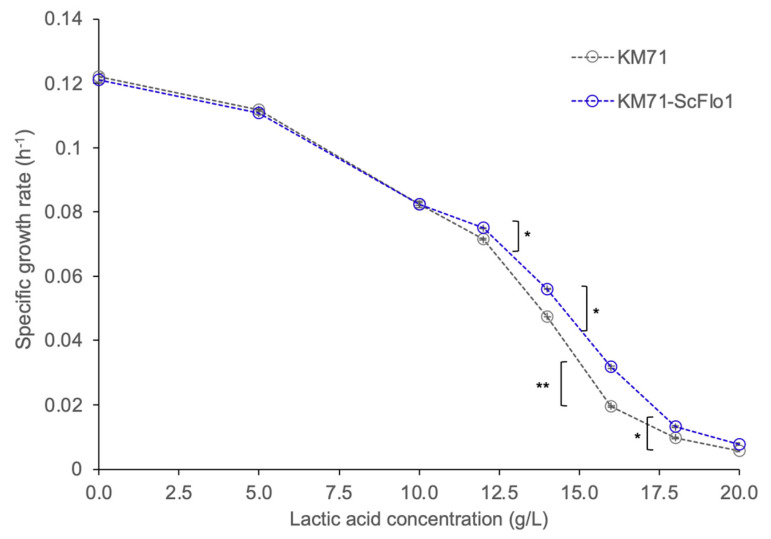
Effects of various lactic acid concentrations in YPD medium (liquid culture) on specific growth rates of *P. pastoris* with or without *ScFLO1* overexpression. Results are the mean of three biological replicates (*n* = 3). Error bars represent the standard deviation from three replicate experiments. * *p* < 0.05, ** *p* < 0.01, two-tail, unpaired, heteroscedastic Student’s *t*-test.

To confer the flocculation phenotype to *P. pastoris*, we heterologously expressed the *ScFLO1* gene cloned from *S. cerevisiae* S288C in *P. pastoris* using the constitutive glyceraldehyde-3-phosphate dehydrogenase promoter (P_GAP_). The resulting strain (KM71-ScFlo1) exhibited a flocculation phenotype when grown in a synthetic defined medium (Figure 1). We next tested KM71-ScFlo1’s tolerance to lactic acid by growing the strain and the non-flocculating control strain (KM71) in a medium containing lactic acid at concentrations ranging from 0 to 20 g/L (Table S3, Figure 2 and Figure 3). Increasing lactic acid concentrations led to lower specific growth rates for both the control and flocculation strains. KM71’s specific growth rates in the rich medium decreased as the concentration of lactic acid in the medium increased, from 0.1221 ± 0.0011 h^−1^ (no lactic acid in the medium) to 0.1119 ± 0.0002 h^−1^ (5 g/L lactic acid) and 0.0825 ± 0.0011 h^−1^ (10 g/L lactic acid). Likewise, KM71-ScFlo1’s specific growth rates in the rich medium decreased from 0.1211 ± 0.0011 h^−1^ (no lactic acid) to 0.1108 ± 0.0006 h^−1^ (5 g/L) and 0.0825 ± 0.0004 h^−1^ (10 g/L). While flocculation did not improve the yeast’s specific growth rates at these concentrations (below 10 g/L) of lactic acid, it appeared to have shortened the lag phase slightly. The positive effects of flocculation on yeast’s tolerance to lactic acid became more evident at higher concentrations (above 10 g/L) of lactic acid, with the biggest improvement in specific growth rate observed at 16 g/L lactic acid (0.0318 ± 0.0004 h^−1^ for KM71-ScFlo1 vs. 0.0195 ± 0.0003 h^−1^ for the control strain, a 1.6-fold improvement). However, once the lactic acid concentration in the medium reached 18 g/L and above, induced flocculation no longer appeared to be sufficient to help the strain cope with the stress. This resulted in KM71-ScFlo1’s specific growth rate dropping to 0.0078 ± 0.0003 h^−1^, a 1.4-fold increase over the control strain’s value of 0.0057 ± 0.0002 h^−1^. Overall, our results indicated that flocculation improved *P. pastoris*’ tolerance to lactic acid. Moreover, induced flocculation could be a viable strategy to increase lactic acid production in *P. pastoris*.

### 3.2. Expression of S. cerevisiae Flocculation Protein Flo1 in P. pastoris Improves d-Lactic Acid Production

**Figure 4:**
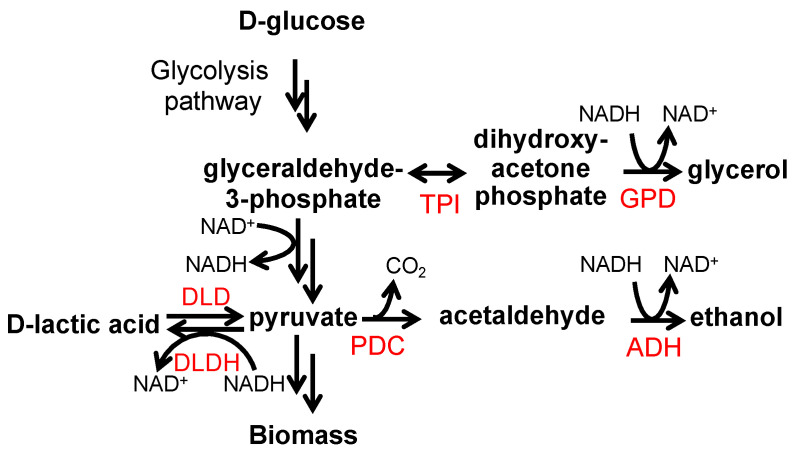
Production of d-lactic acid from glucose. The expression of a d-lactate dehydrogenase (DLDH) enzyme from lactic acid bacteria results in the production of d-lactic acid from pyruvate. By decreasing the production of ethanol and glycerol and limiting the degradation of d-lactic acid, the amount and yield of d-lactic acid can be increased. DLDH, lactic acid bacteria d-lactate dehydrogenase; DLD, endogenous d-lactate dehydrogenase; ADH, alcohol dehydrogenases; PDC, pyruvate decarboxylase; TPI, triosephosphate isomerase; and GPD, glycerol-3-phosphate dehydrogenases.

**Figure 5:**
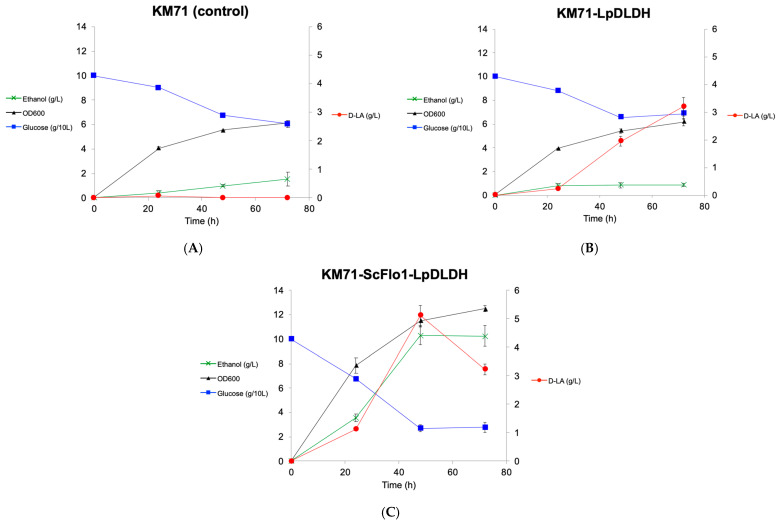
d-lactic acid production in the engineered *P. pastoris* strains: KM71 (control) (**A**), KM71-LpDLDH (**B**), and KM71-ScFlo1-LpDLDH (**C**). Values represent the mean of three biological replicates ± standard deviation (*n* = 3).

Our study of lactic acid tolerance indicated that induced flocculation could improve *P. pastoris’* tolerance to lactic acid at a specific range of concentrations, and suggested that flocculation could potentially increase lactic acid production in *P. pastoris*. To test this hypothesis, we expressed the d-lactate dehydrogenase enzyme from the natural lactic acid producer *Leuconostoc pseudomesenteroides* (*Lp*DLDH) in strain KM71-ScFlo1 using the constitutive P_GAP_ promoter (Figure 4). *Lp*DLDH converts pyruvate to d-lactic acid and has previously been shown to exhibit robust activity in *S. cerevisiae* [2], but its expression in *P. pastoris* had not been demonstrated. The resulting strain KM71-ScFlo1-LpDLDH produced d-lactic acid at a titer of 5.12 ± 0.35 g/L in 48 h (Figure 5). The d-lactic acid titer decreased to 3.23 ± 0.19 g/L at the 72 h time point, possibly due to the d-lactic acid being converted back to pyruvate by an endogenous lactate dehydrogenase enzyme, a phenomenon also observed in engineered d-lactic acid production in other yeast species such as *S. cerevisiae* and *Kluyveromyces marxianus* [2,50]. In comparison, the control strain overexpressing *Lp*DLDH without the flocculation gene produced d-lactic acid at titers of 1.95 ± 0.18 g/L and 3.20 ± 0.33 g/L, at 48 h and 72 h, respectively, and the control strain KM71, which lacks *Lp*DLDH expression, did not produce detectable levels of d-lactic acid.

In addition to d-lactic acid production, we also examined the strains’ ethanol production and glucose consumption. KM71-ScFlo1-LpDLDH produced the highest amounts of ethanol (10.26 ± 0.83 g/L compared to 0.88 ± 0.14 g/L and 1.54 ± 0.56 g/L for KM71-LpDLDH and KM71, respectively) and was the most efficient at consuming glucose (72.3% of glucose consumed compared to 31.3% and 39.5% for KM71-LpDLDH and KM71, respectively). The significant accumulation of ethanol observed in KM71-ScFlo1-LpDLDH is notable since *P. pastoris* is not considered a Crabtree-positive yeast, and therefore is not known for ethanol production under high glucose consumption [51,52]. The control strain KM71 and the strain lacking *ScFLO1* expression produced low amounts of ethanol (9–15% of the amounts observed in KM71-ScFlo1-LpDLDH). The fact that KM71-ScFlo1-LpDLDH produced large amounts of ethanol indicates that enhanced d-lactic acid production may have resulted in imbalances in redox cofactors, leading the cells to consume excess NADH by producing ethanol. To lower ethanol production and enhance d-lactic acid production, several approaches could be considered, such as decreasing the expression of the pyruvate decarboxylase enzyme or increasing the expression of the d-lactate dehydrogenase enzyme to outcompete the endogenous alcohol dehydrogenase enzyme that is responsible for ethanol production.

### 3.3. Transcriptomic Analysis of Engineered Strains

**Figure 6:**
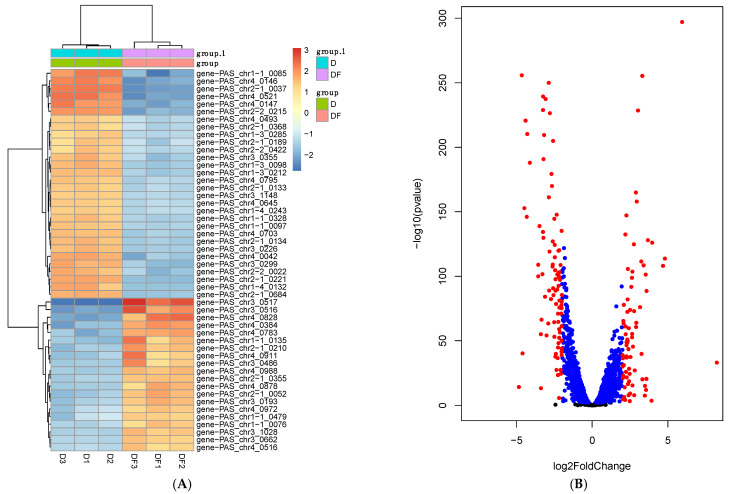
Gene expression heatmap (**A**) and a volcano plot (**B**) of d-lactic acid-producing *Pichia pastoris* with (DF1-3) or without (D1-3) *ScFLO1* overexpression.

To better understand the mechanism behind the improved tolerance to lactic acid of the flocculated yeast strain, we performed a comparative transcriptomic analysis of KM71-ScFlo1-LpDLDH and KM71-LpDLDH. We compared the RNA abundance between the two yeast strains. Using normalized log-expression values, we identified genes that displayed significant changes in expression levels. Genes that satisfied a fold change cut-off of 2 (both positive and negative) and a *p*-value of less than 0.05 were determined to be significantly and differentially expressed. A total of 169 differentially expressed genes were identified, with 77 being upregulated and 92 being downregulated (Figure 6 and Supplementary File S1).

Next, we investigated the functional significance of the differentially expressed genes under lactic acid stress. Lactic acid and other weak acids inhibit the growth of microorganisms, and the extent of inhibition depends on the acid’s chemical properties, pH, and concentration [26,53]. When the pH falls below the pKa of the weak acid, the acid exists primarily in its undissociated and lipophilic form, which can readily penetrate the cell membrane. Once inside, the acid dissociates and accumulates as anions in the cytosol, causing the permeabilization of cellular membranes, the perturbation of membrane proteins, an influx of ions, and the stimulation of proton imports [54,55,56]. The accumulation of anions in the cytosol results in disruption of the electron transport chain, leading to decreased ATP levels and increased reactive oxygen species levels. Additionally, the perturbation of the plasma membrane leads to the influx of protons and intracellular acidification, impacting cellular processes such as synthesis of nucleic acids and other metabolic processes. Finally, the weak acid also binds to metal ions in the growth medium, reducing their availability.

Previous studies have shown that *S. cerevisiae*’s response to weak acids is complex and varies based on the type of acid, with cell-wall function, protein folding, lipid metabolism, and multidrug resistance being the consistently affected biological functions [26,57,58]. The response to weak acids depends on the acid’s lipophilicity, with *TPO3* being the only gene induced in response to all weak acids [57]. This gene codes for a H^+^-antiporter involved in multidrug resistance. The response to hydrophilic acids, including lactic acid, is controlled by the transcription factor Haa1 and the Haa1-regulated genes [55,57,59]. At a low pH, Haa1 localizes to the nucleus and activates Haa1 target genes *YGP1*, *GPG1*, and *SPI1* [60]. At pH 5 and above, when lactic acid exists mainly in the dissociated form (lactate), the transcription factor Aft1 is upregulated, resulting in the increased expression of genes involved in *S. cerevisiae*’s iron metabolism, such as *FIT2*, *ARN1*, and *ARN2* [61].

Based on previous works on lactic acid tolerance in the yeast *S. cerevisiae*, we focused on the upregulation and downregulation of genes involved in transport, iron metabolism, zinc metabolism, and cell-wall rigidity (Table S2). For transport, these are genes that encode: (1) a lactate transporter that is a homolog of *S. cerevisiae* JEN1 (2.26-fold upregulation); (2) a putative transmembrane protein involved in the export of ammonia (2.18-fold upregulation); (3) a hypothetical protein that is a homolog of *S. cerevisiae* ADY2 (2.49-fold upregulation); (4) a putative channel-like protein that is a homolog of *S. cerevisiae* FPS1 (2.19-fold upregulation); (5) an acetate transporter (2.44-fold upregulation); and (6) a plasma membrane H^+^-ATPase (2.06-fold upregulation). For iron metabolism, these are genes that encode: (1) a ferric reductase (6.81-fold upregulation); (2) a Ferro-O2-oxidoreductase (3.80-fold upregulation); (3) a low-affinity Fe(II) transporter of the plasma membrane (3.85-fold upregulation); and (4) a Ferrioxamine B transporter (7.70-fold upregulation). For zinc metabolism, these are genes that encode: (1) a high-affinity zinc transporter of the plasma membrane (27.98-fold upregulation); and (2) a low-affinity zinc transporter of the plasma membrane (6.31-fold upregulation). Finally, for cell wall rigidity, we found a probable catalytic subunit of a mannosylinositol phosphorylceramide (MIPC) synthase (a homolog of *S. cerevisiae* SUR1; 4.09-fold upregulation) and probable monocarboxylate permease proteins (homologs of the *S. cerevisiae* ESBP6; 4–11-fold repression).

Our results indicate that *P. pastoris* and *S. cerevisiae* share many cellular responses to lactic acid stress, including the upregulation of genes involved in lactate transport and iron metabolism [26]. In *S. cerevisiae*, the lactate/H^+^ symporter Jen1 is involved in the transport of lactic acid, as well as other carboxylic acids such as pyruvate and acetate [62,63]. Previous studies have shown that *JEN1* is highly expressed in *S. cerevisiae* cells grown in high concentrations of lactic acid [64,65]. Interestingly, even though Jen1 was initially thought to only transport lactate into the cells, later research suggests it can also export lactate [66,67]. The overexpression of *JEN1* increases lactic acid production in *S. cerevisiae* [66]. Like Jen1, the proton–anion symporter Ady2 transports lactic acid as well as acetate, pyruvate, formate, propionate, and ammonia in *S. cerevisiae* [68,69,70]. The constitutive expression of *ADY2* in *S. cerevisiae* resulted in higher extracellular lactic acid production compared to strains with *ADY2* deletion [69]. In contrast, another study found that *ADY2* deletion improved the growth of *S. cerevisiae* under acetic acid stress, as well as with hydrogen peroxide and ethanol stress [71].

Iron metabolism also plays an important role in lactic acid tolerance in *S. cerevisiae* [26,61]. Under lactic acid stress, the expression of genes related to iron metabolism and homeostasis changes, and this is controlled by the transcription factor Aft1. The activation of Aft1 leads to changes in the expression levels of iron reductases, iron permeases, ferroxidases, and siderophore transporters [61]. The reason for this change in iron metabolism in response to lactic acid stress is not entirely clear, but it is thought that it could be due to either a reduction in the transport of iron or an increased need for iron within the cell, possibly because of iron’s ability to chelate and neutralize lactate. Altogether, our transcriptomics data provide insight into the gene expression changes in *P. pastoris* under high concentrations of lactic acid. The identification of differentially expressed genes, particularly those involved in lactate transport and iron metabolism, provides strong evidence for the role of these genes in adaptation and tolerance to lactic acid.

### 3.4. Overexpression of Several DE Genes Improved d-Lactic Acid Production

**Figure 7:**
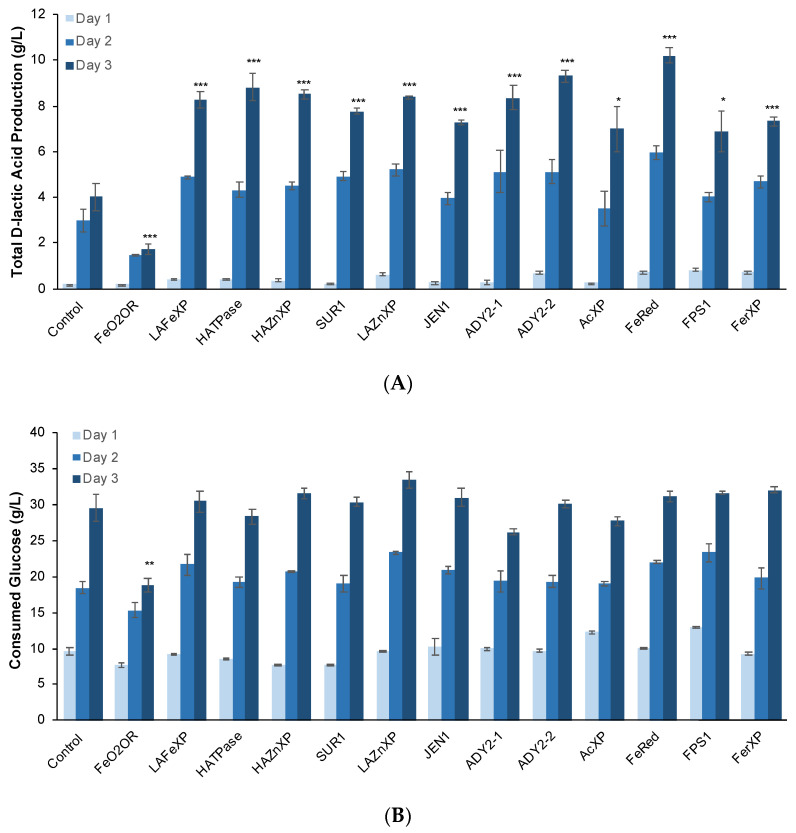
Overexpression of several DE genes improved the production of d-lactic acid. d-lactic acid production (**A**) and glucose consumption (**B**) of engineered *P. pastoris* strains overexpressing selected DE genes identified. Results are the mean of three biological replicates (*n* = 3). Error bars represent the standard deviation. * *p* < 0.05, ** *p* < 0.01, *** *p* < 0.001, two-tail, unpaired, heteroscedastic Student’s *t*-test. Statistical significance is shown for Day 3 results relative to control.

**Figure 8:**
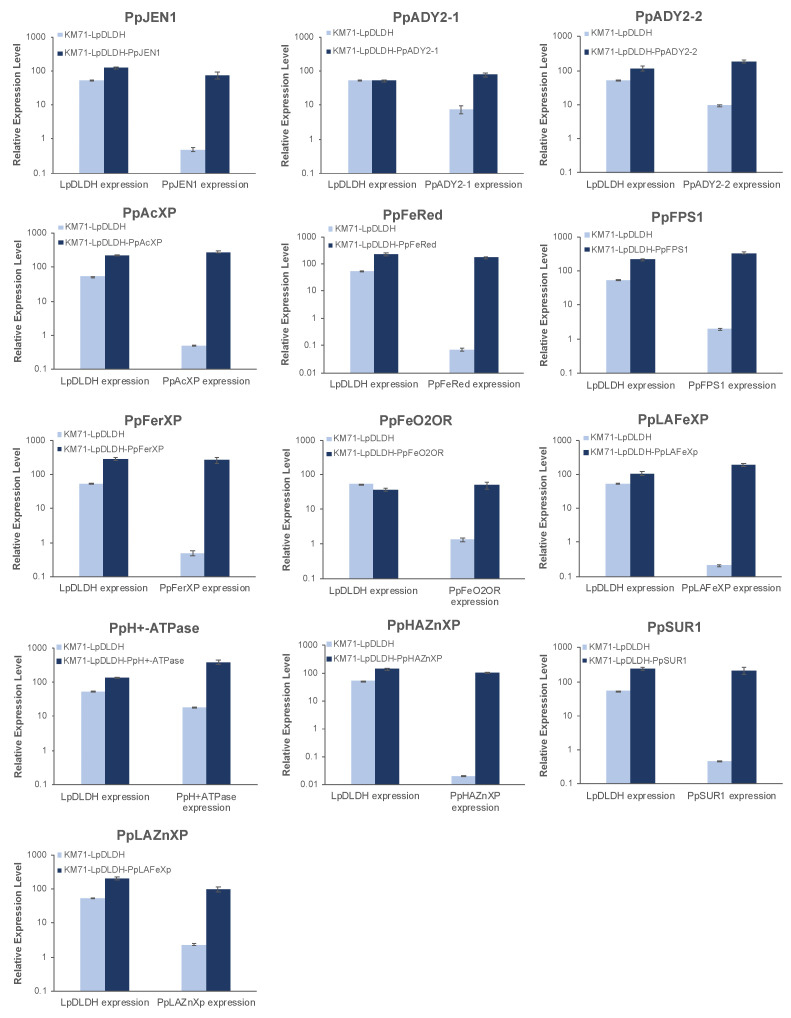
RT-PCR analysis of selected DE genes including *PpJEN1*, *PpADY2-1*, *PpADY2-2*, *PpFeRed*, *PpAcXP*, *PpLAZnXp*, *PpHAZnXP*, *PpFPS1*, *PpFerXP*, *PpSUR1*, *PpH^+^ATPase*, *PpFeO2OR*, *PpLAFeXp*, and *LpDLDH* in engineered yeast. Real-time PCR was performed in three biological replicates. Values represent the mean of three biological replicates ± standard deviation (*n* = 3).

To better understand whether the increase in the expression of these genes affected d-lactic acid production, we overexpressed the individual genes using the constitutive P_GAP_ promoter in the strain KM71-LpDLDH, which overexpresses the enzyme *Lp*DLDH from *L. pseudomesenteroides*. We observed improved d-lactic acid production in several strains (Figure 7 and Figure 8). In particular, the strain that overexpressed the ferric reductase (*Pp*FerRed) had the highest d-lactic acid titer at 10.2 ± 0.3 g/L (with a yield of 0.33 g/g consumed glucose), which was a 2.5-fold improvement over the control strain. Other strains that had significant improvements in d-lactic acid production were those that overexpressed the plasma membrane H^+^-ATPase, the high-affinity zinc transporter, ADY2 homologs, the low-affinity zinc transporter, and the low-affinity iron transporter. Interestingly, overexpression of the Ferro-O2-oxidoreductase resulted in a significant decrease in d-lactic acid production to 1.7 ± 0.2 g/L (with a yield of 0.09 g/g consumed glucose), which was a 58% decrease in titer compared to the control strain.

## 4. Conclusions

This study demonstrates an advancement in the efficient microbial production of d-lactic acid. By incorporating a flocculation gene from *S. cerevisiae* (*ScFLO1*) and a d-lactate dehydrogenase gene from *L. pseudomesenteroides* (*LpDLDH*) into *P. pastoris* KM71, we were able to engineer a strain (KM71-ScFlo1-LpDLDH) that demonstrates increased tolerance to d-lactic acid and improved d-lactic acid production at a titer of 5.12 ± 0.35 g/L in 48 h, a 2.6-fold improvement over the control strain lacking *ScFLO1* expression. Our transcriptomics analysis of this strain provided insights into the underlying mechanism of increased tolerance, including the upregulation of genes involved in lactate transport and iron metabolism and the downregulation of genes involved in cell wall rigidity. We further demonstrated that the recombinant strain overexpressing the ferric reductase (*Pp*FerRed) had the highest d-lactic acid titer, at 10.2 ± 0.3 g/L (with a yield of 0.33 g/g consumed glucose), which was a 2.5-fold improvement over the control strain. To our knowledge, this is the highest d-lactic acid production titer reported in the *P. pastoris* system. This research presents a promising solution to the challenge of tolerance to d-lactic acid in microbial production, and holds the potential for further optimization and scaling up in industrial applications of bioplastic production.

## Figures and Tables

**Table 1:** Engineered strains generated in this study.

Strain Name	Overexpressed Genes	Description	References
KM71	None	Laboratory strain	Invitrogen
KM71-ScFlo1	*ScFLO1*	KM71 overexpressing *ScFLO1* from *S. cerevisiae*	This study
KM71-ScFlo1-LpDLDH	*ScFLO1*, *LpDLDH*	KM71 overexpressing *ScFLO1* from *S. cerevisiae* and *LpDLDH* from *Leuconostoc pseudomesenteroides*	This study
KM71-LpDLDH	*LpDLDH*	KM71 overexpressing *LpDLDH* from *L. pseudomesenteroides*	This study
KM71-LpDLDH-PpJEN1	*LpDLDH, PpJEN1*	KM71-LpDLDH overexpressing endogenous *JEN1*	This study
KM71-LpDLDH-PpADY2-1	*LpDLDH, PpADY2-1*	KM71-LpDLDH overexpressing endogenous *ADY2-1*	This study
KM71-LpDLDH-PpADY2-2	*LpDLDH, PpADY2-2*	KM71-LpDLDH overexpressing endogenous *ADY2-2*	This study
KM71-LpDLDH-PpFeRed	*LpDLDH, PpFeRed*	KM71-LpDLDH overexpressing endogenous *FeRed*	This study
KM71-LpDLDH-PpAcXP	*LpDLDH, PpAcXP*	KM71-LpDLDH overexpressing endogenous *AcXP*	This study
KM71-LpDLDH-PpLAZnXP	*LpDLDH, PpLAZnXP*	KM71-LpDLDH overexpressing endogenous *LAZnXP*	This study
KM71-LpDLDH-PpHAZnXP	*LpDLDH, PpHAZnXP*	KM71-LpDLDH overexpressing endogenous *HAZnXP*	This study
KM71-LpDLDH-PpFPS1	*LpDLDH, PpFPS1*	KM71-LpDLDH overexpressing endogenous *FPS1*	This study
KM71-LpDLDH-PpFerXP	*LpDLDH, PpFerXP*	KM71-LpDLDH overexpressing endogenous *FerXP*	This study
KM71-LpDLDH-PpSUR1	*LpDLDH, PpSUR1*	KM71-LpDLDH overexpressing endogenous *SUR1*	This study
KM71-LpDLDH-PpH^+^-ATPase	*LpDLDH, PpH^+^-ATPase*	KM71-LpDLDH overexpressing endogenous *H^+^-ATPase*	This study
KM71-LpDLDH-PpFeO2OR	*LpDLDH, PpFeO2OR*	KM71-LpDLDH overexpressing endogenous *FeO2OR*	This study
KM71-LpDLDH-PpLAFeXp	*LpDLDH, PpLAFeXP*	KM71-LpDLDH overexpressing endogenous *LAFeXP*	This study

## Data Availability

All data generated or analyzed during this study are included in this published article and its Supplementary Information Files. All raw reads used in the transcriptomic analysis were deposited in the NCBI with the access number GSE224837.

## Supplementary Materials

The following supporting information can be downloaded at: https://www.mdpi.com/article/10.3390/jof9040409/s1, Table S1: Primers used in this study; Table S2: Selected differentially expressed genes identified from transcriptomic analysis; Table S3. Effects of various lactic acid concentrations in YPD medium (liquid culture) on specific growth rates of *P. pastoris* with or without ScFLO1 overexpression. Supplementary File S1: Tabulated differentially expressed genes identified from transcriptomic analysis.
