# Supplementary material for: Engineering Flocculation for Improved Tolerance and Production of d-Lactic Acid in Pichia pastoris
Source: J Fungi (Basel). 2023 Mar 27;9(4):409. doi: 10.3390/jof9040409 (PMC10143824; doi:10.3390/jof9040409)
Supplement: Supplementary file 1 [file jof-09-00409-s001.zip › Tables S1-S3.docx]

**Supporting Information for Engineering Flocculation for Improved Tolerance and Production of D-lactic Acid in *Pichia pastoris***

Kittapong Sae-Tang ^1^, Pornsiri Bumrungtham ^1^, Wuttichai Mhuantong ^1^, Verawat Champreda ^1^,
Sutipa Tanapongpipat ^1^, Xin-Qing Zhao ^2^, Chen-Guang Liu ^2^ and Weerawat Runguphan ^1,^*

^1^ National Center for Genetic Engineering and Biotechnology, 113 Thailand Science Park, Paholyothin Road, Klong 1, Klong Luang, Pathum Thani 12120, Thailand

^2^ State Key Laboratory of Microbial Metabolism, Joint International Research Laboratory of Metabolic &
Developmental Sciences, School of Life Sciences and Biotechnology, Shanghai Jiao Tong University,
Shanghai 200240, China

***** Correspondence: weerawat.run@biotec.or.th

**Table S1. Primers used in this study**

| **Primer Name** | **Sequence (5’ to 3’)** |
| --- | --- |
| Flo1-hom-F | CCGTCTCGGATCGGTACCTCGAGCCGCGGCGGCCGCGAAACGATGACAATGCCTCATCGC |
| Flo1-hom-R | TCAGATCCTCTTCTGAGATGAGTTTTTGTTCGGGCCCTTAAATAATTGCCAGCAATAAGG |
| ScFlo1-5'Seq-R | TAATAGTAGCCAGCGTAC |
| ScFlo1-3'Seq-F | TTAGCGGCGTCACAACAG |
| LpDLDH-F | ATATGGTACCGAAACGATGAAGATCTTCGCTTATGG |
| LpDLDH-R | ATATGCGGCCGCTTAGTACTTAACAGCAATAGC |
| PpJEN1_EcoRI-F | ATAGAATTCATGTCGCATTCAATCCATTC |
| PpJEN1_NotI-R | ATAGCGGCCGCTTACTTATTTCCTTCAAAAGC |
| PpADY2-2_EcoRI-F | ATAGAATTCATGTCTACTCATCAAGATATC |
| PpADY2-2_NotI-R | ATAGCGGCCGCTTAGACATAAAAACCTGCTTG |
| PpADY2-1_EcoRI-F | ATAGAATTCATGGCTGATAATTATAGTATTAAG |
| PpADY2-1_NotI-R | ATAGCGGCCGCCTATTTGGTTCGGAGACC |
| PpFps1_EcoRI-F | ATAGAATTCATGTCATATTCAAAACCACAA |
| PpFps1_NotI-R | ATAGCGGCCGCCTAGGCAATAGTTGGAGC |
| PpAcXp_EcoRI-F | ATAGAATTCATGTCCGAATCTCCGAAT |
| PpAcXp_NotI-R | ATAGCGGCCGCTTAAATAATATAGTTCTTGATATAGA |
| PpFeRed_EcoRI-F | ATAGAATTCATGCTACAATTTATTCCTGTG |
| PpFeRed_NotI-R | ATAGCGGCCGCTTACCATAACTCCAATTCTTC |
| PpFerXp_EcoRI-F | ATAGAATTCATGTCTGCTGAATCTGTG |
| PpFerXp_NotI-R | ATAGCGGCCGCTCAAAGTTTGAAAAGGTTCAT |
| PpLAFeXp_EcoRI-F | ATAGAATTCATGGGCTTCTGGAGAAAT |
| PpLAFeXp_NotI-R | ATAGCGGCCGCCTAAAGCTGATCTACATACTG |
| PpFeO2OR_EcoRI-F | ATAGAATTCATGTTTGTATTCGAACCAG |
| PpFeO2OR_NotI-R | ATAGCGGCCGCTCAGTTCAATAACTCAGTTG |
| PpLAZnXp_EcoRI-F | ATAGAATTCATGTTATTCCCTAGAGAAACC |
| PpLAZnXp_NotI-R | ATAGCGGCCGCTTAGGCCCATTTTCCAAG |
| PpHATPase_XhoI-F | ATACTCGAGATGTCCGCTGAAGAGCCA |
| PpHATPase_NotI-R | ATAGCGGCCGCTTAACCAGACTTCTCGTGCTG |
| PpSUR1_EcoRI-F | ATAGAATTCATGAAAACAGAGCTTAAGATC |
| PpSUR1_NotI-R | ATAGCGGCCGCCTACATGGGTTGGTTCAT |
| PpHAZnXp_EcoRI-F | ATAGAATTCATGAATTTGAAAACTTGGATTACT |
| PpHAZnXp-hom1-R | GCTGGCTTCAGCTCCAAGGGAAACAAATTGGACT |
| PpHAZnXp-hom2-F | CCAATTTGTTTCCCTTGGAGCTGAAGCCAGCAG |
| PpHAZnXp_NotI-R | ATAGCGGCCGCTTATGCCCATTTACCAAGCACAC |

**Table S2. Selected differentially expressed genes identified from transcriptomic analysis**

| **Gene ID** | **Log2FoldChange** | **P_adj_** | **Genbank Accession** | **Product** |
| --- | --- | --- | --- | --- |
| **Upregulated genes** | | | | |
| **Transport** | | | | |
| gene-PAS_chr4_0836 | 0.387376818 | 0.02229365 | XM_002494240.1 | Polyamine transport protein, recognizes spermine, putrescine, and spermidine (homolog to *ScTPO2/ScTPO3*) |
| gene-PAS_chr3_0440 | 1.175922768 | 8.07E-15 | XM_002492622.1 | Lactate transporter (homolog of *ScJEN1*) |
| gene-PAS_chr1-1_0378 | 1.121604232 | 3.38E-07 | XM_002489992.1 | Putative transmembrane protein involved in export of ammonia (homolog of *ScADY2*) |
| gene-PAS_chr1-1_0418 | 0.80951578 | 0.00092879 | XM_002490032.1 | Acetate transporter required for normal sporulation (homolog of *ScADY2*) |
| gene-PAS_chr2-1_0874 | 1.314475951 | 1.41E-11 | XM_002491529.1 | hypothetical protein (homolog of *ScADY2* and *ScATO2/ScATO3*) |
| gene-PAS_chr4_0832 | 0.776259912 | 5.87E-08 | XM_002494235.1 | Plasma membrane ATP-binding cassette (ABC) transporter (homolog of *ScPDR12*) |
| gene-PAS_chr4_0784 | 1.131804182 | 3.51E-18 | XM_002494184.1 | Putative channel-like protein (homolog of *ScFps1*) |
| gene-PAS_chr2-1_0649 | 1.286738148 | 4.01E-14 | XM_002491528.1 | Acetate transporter required for normal sporulation |
| **Iron metabolism** | | | | |
| gene-PAS_chr4_0240 | 2.768506107 | 5.12E-72 | XM_002493603.1 | Ferric reductase, reduces siderophore-bound iron prior to uptake by transporters |
| gene-PAS_chr3_0662 | 2.945776706 | 2.82E-156 | XM_002492843.1 | Ferrioxamine B transporter |
| gene-PAS_chr2-2_0009 | 1.945055952 | 2.69E-07 | XM_002492163.1 | Low-affinity Fe(II) transporter of the plasma membrane |
| gene-PAS_chr2-1_0787 | 1.926646843 | 5.48E-48 | XM_002491680.1 | Ferro-O2-oxidoreductase |
| **Zinc metabolism** | | | | |
| gene-PAS_chr3_0516 | 4.806211183 | 2.44E-112 | XM_002492699.1 | High-affinity zinc transporter of the plasma membrane |
| gene-PAS_chr4_0516 | 2.657951151 | 2.52E-102 | XM_002493905.1 | Low-affinity zinc transporter of the plasma membrane |
| **H+-ATPase** |  |  |  |  |
| gene-PAS_chr1-1_0002 | 1.043159543 | 4.61E-23 | XM_002489588.1 | Plasma membrane H+-ATPase, pumps protons out of the cell |
| **Cell wall rigidity** | | | | |
| gene-PAS_chr1-3_0218 | 2.020911229 | 9.73E-21 | XM_002489517.1 | Probable catalytic subunit of a mannosylinositol phosphorylceramide (MIPC) synthase (homolog of ScSUR1) |
| **Downregulated genes** | | | | |
| **Transporter** | | | | |
| gene-PAS_chr1-1_0398 | -2.022988078 | 2.64E-38 | XM_002490011.1 | Plasma membrane ATP binding cassette (ABC) transporter (Homolog of ScPDR12) |
| gene-PAS_chr1-4_0431 | -2.357365091 | 5.67E-27 | XM_002490517.1 | Plasma membrane multidrug transporter of the major facilitator superfamily (homolog to ScTPO2/TPO3) |
| gene-PAS_chr1-3_0215 | -1.003365594 | 6.09E-08 | XM_002489514.1 | Polyamine transport protein specific for spermine (homolog for ScTPO2/TPO3) |
| gene-PAS_chr2-1_0309 | -2.345279323 | 3.79E-146 | XM_002491159.1 | Protein with similarity to mammalian monocarboxylate permeases (homolog to ScESBP6) |
| **Cell wall rigidity** | | | | |
| gene-PAS_chr3_0278 | -2.016503103 | 2.65E-49 | XM_002492451.1 | Protein with similarity to monocarboxylate permeases (Homolog of ScESBP6) |
| gene-PAS_chr2-1_0309 | -2.345279323 | 3.79E-146 | XM_002491159.1 | Protein with similarity to mammalian monocarboxylate permeases (homolog to ScESBP6) |
| gene-PAS_chr1-4_0132 | -3.481877776 | 2.26E-137 | XM_002490196.1 | Protein with similarity to mammalian monocarboxylate permease (Homolog of ScESBP6) |

**Table S3. Effects of various lactic acid concentrations in YPD medium (liquid culture) on specific growth rates of *P. pastoris* with or without *ScFLO1* overexpression.**

| **Lactic acid concentration (g/L)** | **Specific growth rate (h^-1^)** | |
| --- | --- | --- |
|  | **KM71** | **KM71-ScFlo1** |
| 0 | 0.1221 ± 0.0011 | 0.1211 ± 0.0011 |
| 5 | 0.1119 ± 0.0002 | 0.1108 ± 0.0006 |
| 10 | 0.0825 ± 0.0011 | 0.0825 ± 0.0004 |
| 12 | 0.0715 ± 0.0003 | 0.0751 ± 0.0002 |
| 14 | 0.0475 ± 0.0000 | 0.0561 ± 0.0002 |
| 16 | 0.0195 ± 0.0003 | 0.0318 ± 0.0004 |
| 18 | 0.0097 ± 0.0003 | 0.0132 ± 0.0003 |
| 20 | 0.0057 ± 0.0002 | 0.0078 ± 0.0003 |
