# Supplementary material for: Engineering Flocculation for Improved Tolerance and Production of d-Lactic Acid in Pichia pastoris
Source: J Fungi (Basel). 2023 Mar 27;9(4):409. doi: 10.3390/jof9040409 (PMC10143824; doi:10.3390/jof9040409)
Supplement: Supplementary file 1 [file jof-09-00409-s001.zip › Tables S1-S3.pdf]

Supporting Information for Engineering Flocculation for Improved Tolerance and Production of D-lactic Acid in *Pichia pastoris*

Kittapong Sae-Tang [1], Pornsiri Bumrungtham [1], Wuttichai Mhuantong [1], Verawat Champreda [1], Sutipa Tanapongpipat [1], Xin-Qing Zhao [2], Chen-Guang Liu [2] and Weerawat Runguphan [1,*]

[1] National Center for Genetic Engineering and Biotechnology, 113 Thailand Science Park, Paholyothin Road, Klong 1, Klong Luang, Pathum Thani 12120, Thailand
[2] State Key Laboratory of Microbial Metabolism, Joint International Research Laboratory of Metabolic & Developmental Sciences, School of Life Sciences and Biotechnology, Shanghai Jiao Tong University, Shanghai 200240, China
[*] Correspondence: weerawat.run@biotec.or.th

Table S1. Primers used in this study

Primer Name	Sequence (5' to 3')
Flo1-hom-F	CCGTCTCGGATCGGTACCTCGAGCCGCGGCGGCCGCGAAACGATGACAATGCCTCATCGC
Flo1-hom-R	TCAGATCCTCTTCTGAGATGAGTTTTTGTTCGGGCCCTTAAATAATTGCCAGCAATAAGG
ScFlo1-5'Seq-R	TAATAGTAGCCAGCGTAC
ScFlo1-3'Seq-F	TTAGCGGCGTCACAACAG
LpDLDH-F	ATATGGTACCGAAACGATGAAGATCTTCGCTTATGG
LpDLDH-R	ATATGCGGCCGCTTAGTACTTAACAGCAATAGC
PpJEN1_EcoRI-F	ATAGAATTCATGTCGCATTCAATCCATTC
PpJEN1_NotI-R	ATAGCGGCCGCTTACTTATTTCCTTCAAAAGC
PpADY2-2_EcoRI-F	ATAGAATTCATGTCTACTCATCAAGATATC

PpADY2-2_NotI-R	ATAGCGGCCGCTTAGACATAAAAACCTGCTTG
PpADY2-1_EcoRI-F	ATAGAATTCATGGCTGATAATTATAGTATTAAG
PpADY2-1_NotI-R	ATAGCGGCCGCCTATTTGGTTCGGAGACC
PpFps1_EcoRI-F	ATAGAATTCATGTCATATTCAAAACCACAA
PpFps1_NotI-R	ATAGCGGCCGCCTAGGCAATAGTTGGAGC
PpAcXp_EcoRI-F	ATAGAATTCATGTCCGAATCTCCGAAT
PpAcXp_NotI-R	ATAGCGGCCGCTTAAATAATATAGTTCTTGATATAGA
PpFeRed_EcoRI-F	ATAGAATTCATGCTACAATTTATTCCTGTG
PpFeRed_NotI-R	ATAGCGGCCGCTTACCATAACTCCAATTCTTC
PpFerXp_EcoRI-F	ATAGAATTCATGTCTGCTGAATCTGTG
PpFerXp_NotI-R	ATAGCGGCCGCTCAAAGTTTGAAAAGGTTCAT
PpLAFeXp_EcoRI-F	ATAGAATTCATGGGCTTCTGGAGAAAT
PpLAFeXp_NotI-R	ATAGCGGCCGCCTAAAGCTGATCTACATACTG
PpFeO2OR_EcoRI-F	ATAGAATTCATGTTTGTATTCGAACCAG
PpFeO2OR_NotI-R	ATAGCGGCCGCTCAGTTCAATAACTCAGTTG
PpLAZnXp_EcoRI-F	ATAGAATTCATGTTATTCCCTAGAGAAACC
PpLAZnXp_NotI-R	ATAGCGGCCGCTTAGGCCCATTTTCCAAG
PpHATPase_XhoI-F	ATACTCGAGATGTCCGCTGAAGAGCCA
PpHATPase_NotI-R	ATAGCGGCCGCTTAACCAGACTTCTCGTGCTG

PpSUR1_EcoRI-F	ATAGAATTCATGAAAACAGAGCTTAAGATC
PpSUR1_NotI-R	ATAGCGGCCGCCTACATGGGTTGGTTCAT
PpHAZnXp_EcoRI-F	ATAGAATTCATGAATTTGAAAACTTGGATTACT
PpHAZnXp-hom1-R	GCTGGCTTCAGCTCCAAGGGAAACAAATTGGACT
PpHAZnXp-hom2-F	CCAATTTGTTTCCCTTGGAGCTGAAGCCAGCAG
PpHAZnXp_NotI-R	ATAGCGGCCGCTTATGCCCATTTACCAAGCACAC

Table S2. Selected differentially expressed genes identified from transcriptomic analysis

Gene ID	Log2FoldChange	P_{adj}	Genbank Accession	Product
**Upregulated genes**				
**Transport**				
gene-PAS_chr4_0836	0.387376818	0.02229365	XM_002494240.1	Polyamine transport protein, recognizes spermine, putrescine, and spermidine (homolog to *ScTPO2/ScTPO3*)
gene-PAS_chr3_0440	1.175922768	8.07E-15	XM_002492622.1	Lactate transporter (homolog of *ScJEN1*)
gene-PAS_chr1-1_0378	1.121604232	3.38E-07	XM_002489992.1	Putative transmembrane protein involved in export of ammonia (homolog of *ScADY2*)
gene-PAS_chr1-1_0418	0.80951578	0.00092879	XM_002490032.1	Acetate transporter required for normal sporulation (homolog of *ScADY2*)
gene-PAS_chr2-1_0874	1.314475951	1.41E-11	XM_002491529.1	hypothetical protein (homolog of *ScADY2* and *ScATO2/ScATO3*)
gene-PAS_chr4_0832	0.776259912	5.87E-08	XM_002494235.1	Plasma membrane ATP-binding cassette (ABC) transporter (homolog of *ScPDR12*)
gene-PAS_chr4_0784	1.131804182	3.51E-18	XM_002494184.1	Putative channel-like protein (homolog of *ScFps1*)
gene-PAS_chr2-1_0649	1.286738148	4.01E-14	XM_002491528.1	Acetate transporter required for normal sporulation
**Iron metabolism**				

gene-PAS_chr4_0240	2.768506107	5.12E-72	XM_002493603.1	Ferric reductase, reduces siderophore-bound iron prior to uptake by transporters
gene-PAS_chr3_0662	2.945776706	2.82E-156	XM_002492843.1	Ferrioxamine B transporter
gene-PAS_chr2-2_0009	1.945055952	2.69E-07	XM_002492163.1	Low-affinity Fe(II) transporter of the plasma membrane
gene-PAS_chr2-1_0787	1.926646843	5.48E-48	XM_002491680.1	Ferro-O2-oxidoreductase
**Zinc metabolism**				
gene-PAS_chr3_0516	4.806211183	2.44E-112	XM_002492699.1	High-affinity zinc transporter of the plasma membrane
gene-PAS_chr4_0516	2.657951151	2.52E-102	XM_002493905.1	Low-affinity zinc transporter of the plasma membrane
**H+-ATPase**				
gene-PAS_chr1-1_0002	1.043159543	4.61E-23	XM_002489588.1	Plasma membrane H+-ATPase, pumps protons out of the cell
**Cell wall rigidity**				
gene-PAS_chr1-3_0218	2.020911229	9.73E-21	XM_002489517.1	Probable catalytic subunit of a mannosylinositol phosphorylceramide (MIPC) synthase (homolog of ScSUR1)
**Downregulated genes**				
**Transporter**				
gene-PAS_chr1-1_0398	-2.022988078	2.64E-38	XM_002490011.1	Plasma membrane ATP binding cassette (ABC) transporter (Homolog of ScPDR12)
gene-PAS_chr1-4_0431	-2.357365091	5.67E-27	XM_002490517.1	Plasma membrane multidrug transporter of the major facilitator superfamily (homolog to ScTPO2/TPO3)
gene-PAS_chr1-3_0215	-1.003365594	6.09E-08	XM_002489514.1	Polyamine transport protein specific for spermine (homolog for ScTPO2/TPO3)
gene-PAS_chr2-1_0309	-2.345279323	3.79E-146	XM_002491159.1	Protein with similarity to mammalian monocarboxylate permeases (homolog to ScESBP6)
**Cell wall rigidity**				
gene-PAS_chr3_0278	-2.016503103	2.65E-49	XM_002492451.1	Protein with similarity to monocarboxylate permeases (Homolog of ScESBP6)
gene-PAS_chr2-1_0309	-2.345279323	3.79E-146	XM_002491159.1	Protein with similarity to mammalian monocarboxylate permeases (homolog to ScESBP6)
gene-PAS_chr1-4_0132	-3.481877776	2.26E-137	XM_002490196.1	Protein with similarity to mammalian monocarboxylate permease (Homolog of ScESBP6)

Table S3. Effects of various lactic acid concentrations in YPD medium (liquid culture) on specific growth rates of *P. pastoris* with or without *ScFLO1* overexpression.

Lactic acid concentration (g/L)	Specific growth rate (h^{-1})	
	KM71	KM71-ScFlo1
0	0.1221 ± 0.0011	0.1211 ± 0.0011
5	0.1119 ± 0.0002	0.1108 ± 0.0006
10	0.0825 ± 0.0011	0.0825 ± 0.0004
12	0.0715 ± 0.0003	0.0751 ± 0.0002
14	0.0475 ± 0.0000	0.0561 ± 0.0002
16	0.0195 ± 0.0003	0.0318 ± 0.0004
18	0.0097 ± 0.0003	0.0132 ± 0.0003
20	0.0057 ± 0.0002	0.0078 ± 0.0003
